# The VA Rural Interprofessional Faculty Development Initiative: a qualitative evaluation guided by the RE-AIM framework

**DOI:** 10.1186/s12909-025-08549-x

**Published:** 2026-01-10

**Authors:** Sarah Keithly, Paige Perry, Erica J. Ho, Ryan A. Sterling, Soumya Subramaniam, Shaina Coogan, Sarah E. Shirley, George Sayre, Christian Helfrich, Charles Maynard, Catherine P. Kaminetzky, Erin L. Patel, Joseph Chiovaro, Joel Schmidt, Rebecca Stout, Amber Fisher, Edwin S. Wong, Megan Moldestad

**Affiliations:** 1 Center of Innovation for Veteran-Centered and Value-Driven Care, Puget Sound Health Care System, 1660 S Columbian Way, MS-152, Seattle, WA 98108 USA; 2 Department of Rehabilitation Medicine, University of Washington School of Medicine, WA Seattle, USA; 3 VA Eastern Colorado Healthcare System, Aurora, CO USA; 4 School of Public Health, University of Washington, Health Systems and Population Health, Seattle, WA USA; 5 Office of Academic Affiliations, Veterans Health Administration, Washington, DC USA; 6 Division of General Internal Medicine, University of Washington School of Medicine, Seattle, WA USA; 7 Oregon Health and Science University, Portland, OR USA; 8 VA Central Arkansas Healthcare System, Little Rock, AR USA; 9 Boise VA Medical Center, Boise, ID USA; 10 Veterans Integrated Service Network 20, Veterans Health Administration, Washington, DC USA; 11 University of Washington School of Engineering, Human Centered Design and Engineering, Seattle, WA USA

**Keywords:** Health professions education, Faculty development, Interprofessional education, RE-AIM, Program evaluation

## Abstract

**Background:**

The Department of Veterans Affairs (VA) Rural Interprofessional Faculty Development Initiative (RIFDI) is a longitudinal, multimodal training program designed to improve the quality of and capacity for health professions education in rural areas. This study evaluated RIFDI using the Reach, Effectiveness, Adoption, Implementation, and Maintenance (RE-AIM) framework, with the intent of informing future implementation of this and similar faculty development programs.

**Methods:**

In this descriptive qualitative study, we interviewed RIFDI participants from four program cohorts at different stages of completion. We analyzed data using rapid qualitative analysis and synthesized findings along domains corresponding to the RE-AIM framework.

**Results:**

Forty-one participants completed 49 interviews at mid-program (*n* = 21), program end (*n* = 16), and/or post-program (*n* = 12). *Reach:* Most participants learned about and joined RIFDI via nomination by their site’s educational leadership. Participants were motivated to join by the professional-development opportunity and/or their existing involvement or interest in health professions education. *Effectiveness:* Participants reported improved educational expertise, practice, networking, and institutional knowledge. *Adoption:* Participants perceived leadership support, or lack thereof, and institutional barriers influenced program adoption at their site. *Implementation:* RIFDI’s diverse curriculum was largely perceived as successful in developing professional competencies and networks. Program implementation was aided by blended learning, skilled facilitators, and a collaborative learning environment. *Maintenance:* Participants interviewed 12 months or more post-program reported continued use of acquired knowledge and skills, lasting relationships, and professional opportunities.

**Conclusions:**

RIFDI presents a promising model for interprofessional faculty development in distributed healthcare settings. Its curriculum of didactic, experiential, and collaborative learning provides multiple opportunities for teaching knowledge and skills development and building a community of practice. This study emphasized the importance of institutional support and context in fostering program reach and adoption, and the value of interprofessional networks and blended learning in augmenting program effectiveness, implementation, and maintenance.

**Supplementary Information:**

The online version contains supplementary material available at 10.1186/s12909-025-08549-x.

## Background

Over the past decade, the Department of Veterans Affairs (VA) has expanded investment in health professions education as a strategy for building the workforce needed to deliver high-quality care to underserved rural populations [1]. Highly skilled clinician-educators are essential for creating quality learning environments that both attract trainees and prepare them to meet the evolving demands of health care. Substantial evidence demonstrates the effectiveness of faculty development in improving educators’ teaching competence and practice [2–4]. In two systematic literature reviews, Steinert and colleagues identified the key characteristics of effective faculty development interventions [3, 4]. Among these were longitudinal design, variation in instructional methods, germane content, incorporation of experiential learning and educational projects, deliberate community of practice building, opportunities for feedback and reflection, and institutional support. While the importance of these individual features is widely recognized, evaluations of programs that integrate multiple, complementary approaches remain limited. The current study addresses this gap by evaluating an initiative that combines these evidence-based strategies into a single, comprehensive program tailored for rural, interprofessional clinician-educators.

**Fig. 1:**
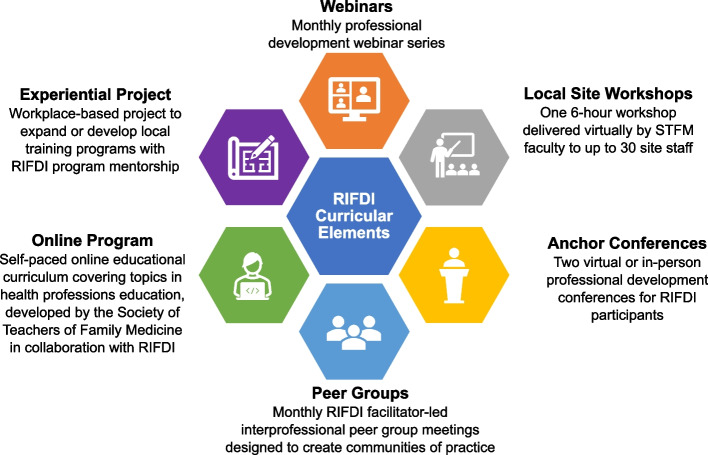
Curricular elements of the VA Rural Interprofessional Faculty Development Initiative (RIFDI)

**Fig. 2:**
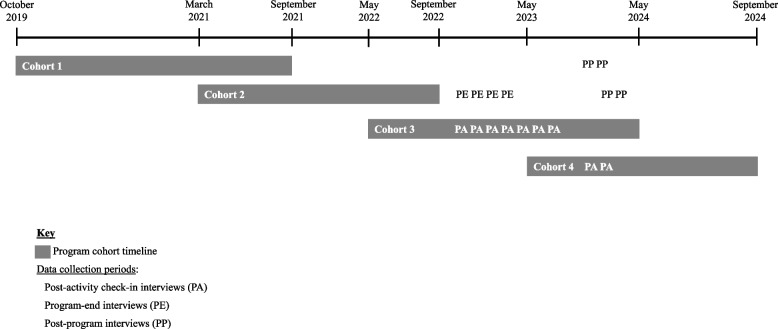
Program and data collection timeline. Footnote: RIFDI’s first cohort was launched in October 2019. Cohorts overlap and range from 16 to 24 months in duration. Qualitative data collection spanned four cohorts and took place between November 2022 and May 2024. Data collection periods are marked as follows: PA: post-activity check-in interviews; PE: program end interviews; PP: post-program interviews (See Table 2 for details on the data collection approach)

In 2019, the VA Office of Academic Affiliations (OAA) and VA Office of Rural Health (ORH) jointly launched the Rural Interprofessional Faculty Development Initiative (RIFDI). RIFDI aims to enhance clinician-educators’ knowledge, skills, and engagement in health professions education, thus building their ability to improve the quality of and capacity for health professions education at VA facilities serving rural Veterans [5]. RIFDI targets clinician-educators from a wide range of professions and health facilities across VA’s national health care system. Its curriculum comprises a unique blend of didactic, experiential, and collaborative learning activities delivered through six program elements, including two educational “anchor” (i.e., program start and completion) conferences, monthly webinars, an online asynchronous training program, monthly interprofessional peer group meetings, a virtual faculty development workshop for participating sites, and a workplace-based experiential project (Fig. 1). The program is delivered over an extended period, ranging from 16 to 24 months across cohorts (Fig. 2). The first half of the program emphasizes didactic learning to develop core teaching competencies and collaborative learning to foster an interprofessional community of practice. In the second half, the focus shifts to leadership development through experiential learning. Program leaders designed RIFDI as a blended learning program, combining face-to-face and online learning activities, to better reach clinician-educators across ≥ 20 VA sites per cohort nationwide. Due to pandemic- and budget-related restrictions, RIFDI implemented a virtual format for Cohorts 2 and 3. Cohorts 1 and 4 held an early in-person conference as originally designed.

This study evaluated the impact of the RIFDI program using the Reach, Effectiveness, Adoption, Implementation, and Maintenance (RE-AIM) framework [6]. The benefits of using the RE-AIM framework are that it facilitates a comprehensive evaluation of programs by addressing organizational- and individual-level factors across five domains influential to real-world impact. It supports translation of research into practice by emphasizing both internal validity (e.g., changes in key outcomes) and external validity (e.g., generalizability of findings) [7]. By using a qualitative approach to RE-AIM, we sought to understand not only whether it works but also how and why it works from the perspective of program participants [8]. Lessons learned will inform continued improvement of the RIFDI program, as well as provide knowledge that translates to faculty development programs across populations and settings.

## Methods

### Study design

This qualitative evaluation was descriptive in nature. Given that RIFDI is an ongoing program, we chose a rapid qualitive approach to data collection and analysis as it enabled sharing of timely feedback to inform real-time decision-making without compromising scientific rigor [9, 10]. Data collection and analysis were grounded in the RE-AIM framework. To operationalize the framework, we developed pragmatic analytic questions structured around each domain, informed by a review of qualitative approaches to RE-AIM [7, 11] and an assessment of what insights could be gained from program participants. Throughout the evaluation, we regularly presented findings to our VA operational partners at OAA and ORH to inform data-responsive program adaptations. Table 1 illustrates how the RE-AIM framework guided the analysis, the results of which identified key areas of program improvement and informed program adaptations.

**Table 1:** Guiding analytic questions, key areas of improvement identified, and related program adaptations

RE-AIM domain	Analytic question(s)	Key area of improvement identified in evaluation	Program adaptation made in response
Reach	What motivated participants to join the program?	A few reported they did not fully understand the program’s purpose and selection process	Began offering information sessions for prospective sites to enhance understanding of the program
Effectiveness	Did participants perceive the program impacted their professional development and educational practice? If so, how?	Some material perceived as less relevant depending on participants’ backgrounds and experience (e.g., too “physician-centric”)	Online learning course was adapted to incorporate the ability to choose electives based on individual learning needs; Tied peer group discussion topics to assigned online module to improve relevance
Adoption	What were the perceived barriers and facilitators to site engagement?	Lack of awareness and support from leadership at some sites hindered the ability to fully participate	Added requirement for supervisor approval for participation
Implementation	What aspects of the program worked well and did not work well, according to participants?	Asynchronous online learning course was time-consuming	Streamlined the online training course to reduce time burden
		Some desired more interaction with participants outside their peer groups	Added collective peer groups to provide more opportunities for interaction with others in the cohort
		Meeting in-person supported engagement and relationship building more than can be achieved in a fully virtual format	Reintroduced the in-person conference post-pandemic, as budget allows
Maintenance	What impacts did participants experience in the long-term?	Several desired more ways to network and stay involved in the program post-program	Created a pathway for alumni to stay involved by serving as peer group facilitators
		A few reported that learnings begin to fade over time	Began inviting alumni to continue attending monthly webinars

### Sampling and recruitment

At the time of recruitment, a total of 178 clinician-educators representing 23 professions and 51 medical facilities nationwide participated in RIFDI across four cohorts. We used a purposive sampling approach to recruit RIFDI participants from the first four program cohorts, each at different stages of completion. The sole exclusion criterion was separation from VA employment at the time of recruitment. Recruitment and data collection occurred in staggered intervals between November 2022 and May 2024 to capture participants at mid-program (Cohorts 3 and 4), program end (Cohort 2), and post-program (Cohorts 1 and 2). Participants could participate in up to two interviews at different program phases. Table 2 summarizes the data collection approach, and Fig. 2 presents the data collection periods relative to the program timeline for each cohort.

**Table 2:** Data collection approach

Interview type and purpose	Data collection	Number of participants
**Post-activity check-ins** to elicit immediate descriptions and perceptions of a program activity	≥ 1 10–15 min. semi-structured interview(s) completed within 1 week of activity date	Cohort 3: 11 participants, 5 of whom completed 2 check-ins Cohort 4: 5 participants
**Program-end interviews** to understand participants’ experiences, perceptions, and impacts associated with the program	1 30–45 min. semi-structured interview completed 3–5 months post-program	16 Cohort 2 graduates
**Post-program interviews** to understand longer-term program impacts	1 20-min. semi-structured interview completed ≥ 24 months post-program (Cohort 1) or ≥ 12 months post-program (Cohort 2)	Cohort 1: 7 graduates Cohort 2: 5 graduates, 3 of whom completed program-end interviews

### Data collection

We conducted virtual, in-depth interviews via Microsoft Teams to better understand clinician-educators’ experiences with and perceptions of RIFDI. Semi-structured interview guides were tailored to the interview type and time point (See Supplemental Material). Areas of focus included most and least beneficial aspects of the program; facilitators and barriers to program completion; perceptions and experiences of interprofessional learning; and professional development. Interviews were recorded and transcribed using Microsoft Teams. To ensure accuracy of the transcription, interviewers compared automated transcripts against the recording and corrected transcription errors.

### Data analysis

We employed a rapid qualitative analysis approach developed by Hamilton and colleagues [10, 12] that combines structured template analysis and matrix analysis to facilitate manifest content analysis. Immediately following interviews, analysts summarized transcripts using a structured template with deductive domains, as well as an “other” domain for inductive findings. To ensure confidentiality, interview summaries excluded information that could be used to identify a specific participant. Summary data was then copied into a matrix to compare responses within and across domains. Lastly, analytic questions along each domain of the RE-AIM framework guided data synthesis (Table 1).

### Rigor

We employed the following strategies to ensure study rigor. Throughout the study, we maintained thorough documentation of our processes to support an audit trail. To minimize interviewer influence, interviewers used grounded probes that incorporated participants’ verbatim language to elicit details and clarify responses [13]. In addition, interviewers and analysts used memoing to reflect on how their backgrounds, views, and experiences might influence the results. The lead analyst reviewed all interview summaries to ensure overall consistency and quality. To minimize influence of VA operational partners responsible for implementing RIFDI, evaluation activities were conducted independently by the evaluation team. Operational partners provided contextual information to support evaluation planning and implementation. To strengthen the validity and credibility of the analysis, the evaluation team met regularly to review preliminary findings, then reviewed findings with operational partners. By gathering input from individuals with diverse backgrounds, roles, and expertise, this process ensured findings accurately reflected both the data and the operational context.

### Results

Forty-one RIFDI participants from 27 VA medical centers and 11 professions completed an interview (Table 3). Of those, eight completed two interviews each for a total of 49 distinct interviews, including 21 post-activity check-ins, 16 program end interviews, and 12 post-program interviews. The following sections present key findings informing each RE-AIM domain. Quotations are labeled with identification numbers, the first digit of which denotes the cohort number followed by a randomly assigned number.

**Table 3:** Characteristics of clinician-educators who participated in evaluation interviews

Characteristics	Interview participants (*N* = 41)^a^
**RIFDI cohort, no. (%)**	
1	7 (17.1%)
2	18 (43.9%)
3	11 (26.8%)
4	5 (12.2%)
**Age, mean (SD)**	46.4 (9.0)
**Sex (female), no. (%)**	31 (75.6%)
**Years of clinical practice experience**	
< 5	4 (9.8%)
5- < 10	9 (22.0%)
10 +	24 (58.5%)
Unknown	4 (9.8%)
**Rurality of participant’s VA medical site, no. (%)**	
Serves < 50% rural Veterans	16 (39.0%)
Serves ≥ 50% rural Veterans	23 (56.1%)
Unknown	2 (4.9%)
**Profession**	
Physician	11 (26.8%)
Psychologist	7 (17.1%)
Nurse Practitioner	6 (14.6%)
Pharmacist	4 (9.8%)
Social Worker	3 (7.3%)
Nurse	2 (4.9%)
Other ^b^	8 (19.5%)

Abbreviations: *no* number, *SD* Standard deviation

^a^Of the 41 participants, eight completed two interviews each for a total of 49 interviews

^b^The eight participants listed as ‘other’ came from five different professional backgrounds, which remain unlisted to protect participant confidentiality, given their limited representation in the RIFDI program overall

### Reach

RIFDI recruitment involved sending a request for proposals to eligible VA facilities. Leadership from selected sites could nominate up to two clinicians to join the program. The most cited reason for joining RIFDI was nomination by leadership, such as the site’s designated education officer, the position in VA responsible for overseeing all health professions education programs within a facility. Some perceived their nomination as an optional invitation to join, which they accepted based on their desire to grow professionally and/or their existing interest or involvement in health professions education. Others perceived their nomination as a directive to join. Despite the different routes to participation, no notable differences were found between these groups in program satisfaction or effectiveness.

*“I kind of got volun-told. Like, ‘Oh, there's this thing, and we don't really know who can take any lead on it for our facility.’ [We] were just kind of thrown into the mix.”* – 2011

Some RIFDI alumni went on to select participants for future cohorts by virtue of their position as the facility’s designated education officer. These individuals described choosing prospective participants mindfully, based on the suitability of the nominee and the educational needs of their facility.

*“The people involved in training now were more handpicked … I was able to select the RIFDI members in what I thought was areas that we needed … People more involved with programs that we were trying to grow.”* – 1011

### Effectiveness

Perceived effectiveness was reflected in self-reported gains in knowledge and skills, which participants attributed in part to the curriculum’s focus on core teaching competencies. Many participants applied their learnings in a variety of ways, such as making changes to their teaching approach or curriculum. Topics repeatedly highlighted as beneficial included giving feedback, learning styles, interprofessional collaboration, and time management. However, some perceived selected material as less relevant depending on participants’ backgrounds. For example, several reported the online training course was too “physician-centric” and therefore less applicable to non-physician educators.

Most participants reported that RIFDI fostered relationships and expanded their interprofessional networks, a benefit that was often attributed to peer groups. Peer groups reinforced learnings and supported participants’ teaching practice by offering a forum for knowledge sharing, reflection, problem-solving, and feedback. Participants repeatedly emphasized the value of networking with peers in other VA sites and professions, as it exposed them to new information, strategies, and resources for working within the VA health care system. By enhancing their institutional knowledge and resources, participants felt more capable of advancing local programs and activities.

“*The small group was great [for] meeting people in other VAs. I think that we got some good resources working with the folks that were in the [site] VA system. So just talking to other people and learning about what they're going through in their system… It definitely helped me, again, have more resources within the VA moving forward.” –* 2040

Participants found that experiential projects built their skills and confidence in leadership, particularly among those with limited prior experience in health professions education program development.

*“I feel better at that management aspect of things that I as a* [profession], *you don't really do a whole lot…I feel like going forward, I would be more confident and more willing to take on projects.”* – 4001

Participants in educational leadership positions, such as training program directors, reported that the experiential projects advanced their work to develop health professions education programs, primarily through the resources, guidance, and feedback gained from their RIFDI peers and faculty.

### Adoption

Participants reflected on their site’s adoption of RIFDI in their reports of facilitators and barriers to full participation in the program. To secure institutional support, RIFDI required site leadership to commit to 10% release time for program activities. Despite this requirement, participants described varying degrees of institutional backing, illustrated by the following two accounts.

*“As far as my supervisor, they were not aware of [RIFDI] … So, it was a little difficult to have the time that I needed to work on the project … Even the Education Director wasn't really familiar with everything that it entailed.” –* 2013

*“My Director of Education*… *was really supportive. She wanted to see me complete this. It's a priority..*. *I did have the backing to complete RIFDI.” –* 2019

Many felt that having this protected time was instrumental in supporting their ability to participate in the program. However, several noted difficulties in truly protecting time due to unsupportive leadership, productivity pressures, and workload. In the first two cohorts, this challenge was exacerbated by the COVID-19 pandemic, which increased clinical demands. Several participants emphasized that patient care took precedence over professional development. Cited challenges to implementing experiential projects included facility leadership turnover, limited space, financial constraints, limited time or interest among the site’s providers, and burdensome institutional procedures.

*“We were passionate about getting [our project] off the ground, but we really couldn't. We were busy putting out fires trying to fill in staffing shortages…patients had to be taken care of, that was our first priority.” –* 2007

Using funding available under the Veterans Access, Choice, and Accountability Act of 2014, OAA awarded infrastructure funding to selected RIFDI sites from Cohorts 1 and 2 to support health professions education at the facility. At the discretion of site leadership, some RIFDI participants used these funds for their experiential projects. Some participants perceived that infrastructure funding incentivized leadership buy-in, which made it easier to pursue RIFDI activities at the site. Site engagement was also facilitated by educational leadership participation in RIFDI. Program alumni who were VA designated education officers supported program recruitment, promoted RIFDI activities, and supported experiential projects at their site.

“*My goal is to develop a [communication channel] for our RIFDI graduates... I keep tabs on them as the [designated education officer], and it helps that I'm a graduate … I did make sure yesterday that everyone in the [department] knew what RIFDI was.”* – 2029

### Implementation

Overall, participants gave highly positive accounts of their time in RIFDI, which they attributed to its emphasis on collaborative learning, multimodal approach, quality curriculum, and committed, knowledgeable program staff. Among the most valued program elements was the in-person conference held in Cohorts 1 and 4. Attendees attributed the success of the conference to the in-person modality, engaging speakers and topics, supportive environment, and use of interactive activities. Meeting in-person was perceived to support deeper relationships by creating an environment that “truly was protected time” and by allowing for more personal interactions than can be achieved in virtual settings.

*“There’s still that power of connecting to people in person… when you’re at a conference … and you’re in the room with the other people, I truly believe there’s a synergy that happens when you’re in that place.” –* 1008

Regardless of modality, the first conference played a foundational role for the program by helping participants get to know each other and the program staff. It also set a welcoming tone, created excitement for the program, and provided knowledge participants could immediately apply in their own teaching.

*“[Conferences are] really helpful. Especially the opening conference… because it gives you a grounding. It's like building a foundation to start building a house on. So, the conferences help provide the foundation for the house. … It also gets you into the sense that you're part of a group that's doing this, which is huge that you're not just out there on your own.”* – 2019

The second conference was held virtually across all cohorts. It was designed to provide an intensive learning experience covering recurring program themes, such as educator skills, leadership skills, and wellness. Participants’ accounts of the second conference were comparatively infrequent and less evocative than the first, suggesting it was less salient to their experience. Feedback on the event largely mirrored that of the other two virtual didactic activities, webinars and workshops. These activities were generally well-received, with participants citing the quality speakers and content; however, many reported that engagement was hindered by distractions and multitasking. Strategies that facilitated virtual engagement included use of interactive exercises, such as online breakout rooms. Participants appreciated the ability to view recorded sessions asynchronously.

Perceptions of the asynchronous online training program varied widely among participants. Participants generally liked that the program was self-paced, incorporated interactive learning activities, and used a microlearning approach in which topics were broken down into “small chunks.” However, some participants found it challenging to stay engaged in asynchronous virtual learning, and several noted that it was time consuming, causing them to fall behind or use personal time to complete coursework.

Participants often attributed the level of effectiveness of their peer group to the RIFDI facilitators. They described effective facilitators as approachable, responsive, knowledgeable, and skilled in moderating discussions. Group meetings were regarded as more successful when they were structured around a topic and guided by discussion questions. Most participants valued the interprofessional makeup of the peer groups because it exposed them to diverse perspectives and approaches to teaching and encouraged “cross-pollination” of knowledge.

*“I do like the [peer] groups being interdisciplinary… it introduces people to professions that they wouldn’t necessarily otherwise collaborate with or understand… you just learn different things from different people.” –* 3013

However, a few found that the differences between the professions limited the group discussions because some information did not apply across programs. These participants expressed a desire to engage more with peers from their own profession to maximize the utility of the discussions.

### Maintenance

In interviews completed roughly 12 to 24 months post-program, most participants reported that they continued to apply the knowledge, skills, and resources gained from RIFDI. Several participants made lasting changes to their teaching approach, such as using feedback techniques and setting expectations. However, a Cohort 1 participant observed that there is a “tapering off effect” over time for some of the learnings. Participants in educational leadership positions shared examples of how they incorporated RIFDI’s learning materials into health professions education programs or shared these resources with other VA clinician-educators.

Several participants reported that they have maintained contact with the professionals they met through RIFDI, and these connections remain a valued source of mutual support and motivation. The potential for long-lasting, productive relationships was exemplified by the accounts of three participants who collaborated on a joint experiential project and continued to meet more than two years after the program.

*“We continue to meet monthly as a group to share resources both for internal to our roles but also cross-collaborate in terms of resources, didactics, and such for different programs … That's been some of the longer lasting results of our project.” –* 1011

However, some participants reported that they maintained limited-to-no contact with their RIFDI colleagues after program end. Several suggested that RIFDI should provide more ways for alumni to network and stay involved.

Some participants perceived that RIFDI contributed to professional growth and job fulfillment. Multiple cited RIFDI as a reason for them obtaining educational leadership positions in their facility, in part by gaining recognition from leadership due to their experiential projects.

*“[The RIFDI project] gave me the visibility in working with the [facility leader]. Then the [senior] position came open and I was approached by executive leadership to throw my hat in the ring for it. Then I was able to get the position, I think largely in part due to the RIFDI program.” –* 2004

For some, the professional development, networks, and recognition gained from their participation in RIFDI opened opportunities to balance their clinical duties with educational work. For example, a participant described how their RIFDI connections were integral to their success in getting more involved in education. The enjoyment and rewards gained as a result reduced their feelings of burnout and compelled them to extend their employment at VA.

*“I stayed [at VA] longer than I was going to because I've enjoyed these last couple of years in the training program so much … It was being involved in the training program, the residency program, at the level that I'm involved now, that really gave me the motivation to stick with it a few extra years.” –* 1039

Other participants gave similar accounts linking RIFDI to improved job satisfaction and motivation. Some expressed that the program helped them recognize the importance of rural health professions education and healthcare. This change in perspective added meaning and purpose to their work. One participant reported that this new perspective motivated them to encourage trainees to practice in rural health settings, and young providers joined the rural facility due in part to their positive training experience.

*“We have a few young providers here who you will never think would come to a quiet rural community like this. And they do. And some of them will tell you, ‘Oh, it's because of when I rotated, I thought it was kind of cool. I learned a lot from this community. I get a chance to see some things that you may not see in in bigger places.’” –* 1027

## Discussion

In this qualitative evaluation, we used RE-AIM as a guiding framework to assess the essential elements of a VA interprofessional faculty development program for rural clinician-educators from the perspectives of program participants. The results suggest that RIFDI has been effective in professionally developing VA clinician-educators by improving teaching competencies and practice, enhancing professional networks, and supporting institutional engagement. Rapid qualitative analysis enabled us to provide actionable insights throughout program implementation, which the RIFDI team used to adapt the program. Insights from this study may inform the design, delivery, and evaluation of faculty development programs both within and outside VA, with the long-term goal of strengthening health workforce capacity, particularly in rural areas.

Our findings are consistent with the literature showing that institutional leadership support is key to the success of faculty development programs in health professions education [3, 14]. In RIFDI, facility leaders influenced program impact across multiple domains but played a critical role in shaping program reach as they were responsible for selecting clinician-educators to participate. Recruiting educational leaders to complete the program emerged as a promising strategy for enhancing program reach. Alumni can leverage their leadership role and program experience to recruit clinician-educators who are best positioned to reap benefits for both themselves and the facility. Further, these leaders may serve as program champions who support participants’ engagement and site activities during the program and beyond.

Our finding that participation in RIFDI was, in some cases, either expected or required has important implications. Mandatory participation may have potential advantages beyond clinician-educators’ professional development, such as fostering a culture that values health professions education and continuous improvement, ensuring compliance with institutional standards, and promoting collaboration among faculty members [4]. In weighing between mandatory or voluntary participation, institutions should carefully consider the unique needs and context of its clinicians to ensure they have the support and flexibility needed for meaningful participation.

In addition to reach, facility leaders strongly influenced adoption, particularly in their willingness to honor protected time for faculty development. While all participants were formally granted protected time, the ability to use that time for training was often limited by lack of leadership support and competing demands. Leaders must weigh the benefits of professional development against the challenges of delivering health care, especially in rural settings, such as staffing shortages and limited resources [15]. Getting leadership buy-in requires effective communication of the benefits a facility stands to gain from faculty development, such as the potential to attract and retain qualified health professionals. We found that helping participants secure grant funding that aligns with an institution’s needs was a powerful incentive for institutional support. Another strategy is to consider offering condensed training options for sites strained for capacity, ideally informed by an evaluation of the tradeoffs between program adoption and effectiveness.

RIFDI’s perceived effectivene**ss** was reflected in participant-reported improvements in their knowledge, skills, teaching practice, and professional networks. Didactic activities considered most relevant to participants’ day-to-day work were the most valued, presenting a challenge to faculty development programs like RIFDI that target clinician-educators who vary widely in profession, role, and level of experience. Interprofessional programs must strike a balance between ensuring the material broadly applies while also retaining value for specific educational contexts. By supplementing didactic learning with experiential learning and collaborative learning approaches, RIFDI provided a means for participants to obtain value from different aspects of the program. For example, experienced clinician-educators with a strong foundation in teaching methods highly valued the ability to access the expertise and resources of RIFDI’s community of practice. This finding adds to the body of evidence showing the effectiveness of faculty development programs that use a multimodal educational approach [3] and support formation of communities of practice [16–19].

Multiple factors contributed to RIFDI’s successful implementation. The program’s curricular elements worked synergistically to enhance professional development and create a community of practice. Holding an early conference, preferably in-person, may support longitudinal programs by providing a protected space for relationship building and by setting the tone for the subsequent program activities, such as webinars and peer groups. While virtual activities posed challenges to engagement, they were valued for their flexibility and ability to connect colleagues across long distances, supporting the assertion that blended learning can afford the benefits of both in-person and online learning modalities [20].

Monthly peer groups provided repeated opportunities for connection within a community of practice. Learning with an interprofessional peer group broadened participants’ perspectives and enhanced their knowledge of the different professions, yet participants also valued opportunities to learn from peers from the same profession as their experience was more applicable. Taken together, these findings suggest that interprofessional faculty development programs should consider ways to incorporate both inter- and intra-professional peer learning.

Regarding maintenance of program impact, participants reported lasting changes to their teaching competencies, practice, and professional relationships. There was some evidence, however, of these benefits fading over time. Offering alumni avenues for continued involvement could help mitigate this effect. For example, starting in Cohort 4, RIFDI invited selected alumni to serve as peer group facilitators, providing them with the opportunity to stay connected with the program and to continue building their skills and networks. Online platforms may prove a useful tool for the larger community to stay involved, as they provide an accessible forum for participants to continue sharing knowledge and resources, as well as a means for the institution to track accomplishments and developments within the community [16].

Long-term impacts reported by program participants provide preliminary evidence that RIFDI has advanced its objective of improving workforce retention. Clinician-educators described ways in which RIFDI positively influenced their career pathways, job satisfaction, and sense of purpose, all shown to be mediators on the pathway between professional development and staff retention [21, 22]. Further, RIFDI motivated some to recruit future health care professionals to rural settings, in part by enhancing their appreciation of the value of health professions education. While these reports are promising, more research is needed to assess the degree to which RIFDI has contributed to attracting and retaining health professionals.

This study has limitations. Participants who agreed to be interviewed may differ from those who did not, which may bias results. In addition, these findings represent cross-sectional snapshots wherein we collected data from multiple cohorts at different stages of program completion. Therefore, the factors driving program success may vary according to differences inherent to the cohorts and program timing. We continue to conduct interviews with current cohorts so that future findings from this evaluation can better distinguish participants’ experiences that are a function of the participants’ cohort or point in program participation. Lastly, this analysis focused on RIFDI’s effectiveness on proximal outcomes, specifically, perceived changes in participants’ professional development and teaching practice. Additional mixed methods analyses are underway to examine more distal outcomes that may result from these changes, including rural educational program development, staff turnover, and trainee presence.

## Conclusions

This comprehensive qualitative evaluation suggests the program has positively impacted participating clinician-educators and reveals factors influential to the program’s success that may help guide future implementation of RIFDI and similar programs. A longitudinal program design employing a mix of didactic, experiential, and collaborative learning approaches is conducive to both learning and community of practice formation. Distributed learning programs may benefit from blended learning to maximize the benefits of in-person and virtual engagement. Gaining leadership support is fostered by recruiting leaders to participate in the program and offering financial incentives to participating sites. More research is needed to determine the degree to which RIFDI and similar programs impact long-term outcomes such as health workforce recruitment and retention, changes to interprofessional collaborative practice, and improvements in patient access and care.

## Supplementary Information

Supplementary Material 1.

## Data Availability

The authors do not have permission to share data collected for VA quality improvement efforts. Selected de-identified data supporting the reported findings are available within the article.
