# Supplementary material for: The VA Rural Interprofessional Faculty Development Initiative: a qualitative evaluation guided by the RE-AIM framework
Source: BMC Med Educ. 2026 Jan 10;26:215. doi: 10.1186/s12909-025-08549-x (PMC12882197; doi:10.1186/s12909-025-08549-x)
Supplement: Supplementary file 1 — Supplementary Material 1. [file 12909_2025_8549_MOESM1_ESM.docx]

**Supplemental Material: Interview Guides**

**Post-Activity Check-In Interview Guide**

**Introduction: First Interview**

Hello [participant name],

My name is [interviewer name] and I am from the [location] VA. (*Also on the call is [second interviewer name*].)

I am (*We are*) part of an evaluation team working with OAA to understand the experiences of staff taking part in the RIFDI program. Our goal is to understand the impact of RIFDI on your professional development and to see how we might improve the program.

We really appreciate your willingness to be interviewed. The call will take approximately 10-15 minutes.

Taking part in this interview is voluntary and your responses will be strictly confidential.

We also want to make clear that even though we’re working with OAA and the RIFDI team, we only present them deidentified findings. In other words, you will not be identified in any reports or presentations.

Last, in order to make sure we capture all the information you share, I’d like to record this call. An audio recording and transcript will be created. The recording and transcript will be transferred to a secure server that only the evaluation team has access to. You may ask us to pause or stop recording at any time.

[*If the participant has their video on, please let them know that the recording will include video unless they turn it off. Give them time to turn off their video if they prefer to record audio only*.]

Is this okay with you?

Before I start recording, do you have any questions for me (*us*)?

[**Hit record button**.] Okay, to confirm, I’m starting the recording with –

- Audio and transcript. Is this okay with you?
- Audio-video and transcript. Is this ok with you?

**Main Questions: First Interview**

1. Tell me about your role at VA.
2. Walk me through how you came to the RIFDI program.
3. Our records show you recently completed a [conference, webinar, site workshop, RIFDI-FIT online module, project, peer group meeting]. Tell me about it.
   1. Tell me what you liked about that [activity], if anything.
   2. Tell me what you did not like about that [activity], if anything.
4. [*Time permitting*]
   1. (*If no other activities mentioned*) Tell me about other activities you’ve done since starting the program.
   2. (*If other activities mentioned*): You mentioned [activity].
      1. Tell me what liked about that [webinar, workshop, other activity], if anything.
      2. Tell me what you did not like about that [webinar, workshop, other activity], if anything.
5. Anything else you’d like to add?

Finally, we may want to follow up with you in the future on other elements of the RIFDI program. Would you mind if we contacted you again for another interview?

**If YES**: Great, thank you. I’ll reach out if and when we want to speak with you again. Thank you again for taking part in today’s call.

**If NO**: No problem, thanks for taking part in today’s call.

**Introduction: Subsequent Interview**

Hello [participant name],

Thanks for agreeing to speak with me (*us*) again. Last time, we talked about your experience with [RIFDI activity]. For this interview, we’re interested in talking about your experience with the [most recent RIFDI activity].

The call will take approximately 10-15 minutes.

Like before, taking part in this interview is voluntary and your responses will be strictly confidential. In order to make sure we capture all the information you share, I’d like to record this call. An audio recording and transcript will be created. The recording and transcript will be transferred to a secure server that only the evaluation team has access to. You may ask us to pause or stop recording at any time.

[*If the participant has their video on, please let them know that the recording will include video unless they turn it off. Give them time to turn off their video if they prefer to record audio only*.]

Is this okay with you?

Before I start recording, do you have any questions for me (*us*)?

[**Hit record button**.] Okay, to confirm, I’m starting the recording with –

- Audio and transcript. Is this okay with you?
- Audio-video and transcript. Is this ok with you?

**Main Questions: Subsequent Interview**

If needed: What activities have you completed?

1. Our records show you recently completed a [conference, webinar, site workshop, RIFDI-FIT online module, project, peer group meeting]. Tell me about it.
   1. Tell me what liked about that [webinar, workshop, other activity], if anything.
   2. Tell me what you did not about that [webinar, workshop, other activity], if anything.
2. [*Time permitting*]
   1. (*If no other activities mentioned*) Tell me about other activities you’ve done since we last spoke.
   2. (*If other activities mentioned*): You mentioned [activity].
      1. Tell me what liked about that [webinar, workshop, other activity], if anything.
      2. Tell me what you did not about that [webinar, workshop, other activity], if anything.
3. Anything else you’d like to add?

**Structured Probes (First and Subsequent Interview)**

*We use structured probes frequently to follow up on any responses to the main questions on the guide. Use* ***verbatim*** *participant language – for example, if they say the program was “hard,” say “tell me more about it being hard,” NOT “tell me more about it being difficult, challenging,” etc.*

*Walk me through ___________.*

*Tell me more about ___________.*

*Give me an example of ___________.*

*Tell me about a time when ___________.*

*What were the benefits of ___________?*

*What were the challenges of ___________?*

*How did you learn about_________?*

*Did you receive any support for __________?*

*What could be improved about __________?*

*Who ___________?*

*When ___________?*

*Where ___________?*

**Program-End Interview Guide**

**Introduction**

Hello [participant name],

My name is [interviewer name] and I am from the [location] VA. (*Also on the call is [second interviewer name*].)

I am (*We are*) part of an evaluation team working with OAA to understand the experiences of staff taking part in the RIFDI program. Our goal is to understand the impact of RIFDI on your professional development and to see how we might improve the program.

We really appreciate your willingness to be interviewed. The call will take approximately 30 – 45 minutes. Does that still work for you?

Taking part in this interview is voluntary and your responses will be strictly confidential.

We also want to make clear that even though we’re working with OAA and the RIFDI team, we only present them deidentified findings. In other words, you will not be identified in any reports or presentations.

Last, in order to make sure we capture all the information you share, I’d like to record this call. An audio recording and transcript will be created. The recording and transcript will be transferred to a secure server that only the evaluation team has access to.

[*If the participant has their video on, please let them know that the recording will include video unless they turn it off. Give them time to turn off their video if they prefer to record audio only*.]

Is this okay with you?

Before I start recording, do you have any questions for me *(us*)?

[**Hit record button**.] Okay, to confirm, I’m starting the recording with –

- Audio and transcript. Is this okay with you?
- Audio-video and transcript. Is this ok with you?

**Main Questions**

1. Tell me about doing the RIFDI program.
   1. IF NEEDED: What is your role at VA?
2. What do you consider some of the most beneficial aspects of the program?
3. What do you consider some of the least beneficial aspects of the program?
4. What, if anything, made completing the program harder?
5. What, if anything, made completing the program easier?
6. Tell me about training with people from other sites and disciplines.
7. I want to come back to the various RIFDI elements. You’ve already talked about [______]. What about the…
   - 1. First and last conferences? (aka: Anchor conferences)
     2. Small peer groups?
     3. Virtual presentations? (aka: Webinars)
     4. Experiential projects?
     5. STFM online modules? (aka: RIFDI-FIT modules)
     6. STFM workshops? (site specific, 2 site cohort members) (aka: Site faculty workshops)
8. How have the skills you gained from RIFDI…
   1. … been applied to your clinical practice and teaching?
   2. … improved the training programs at your facility?
9. Tell me about teaching in a rural setting.

AS NEEDED:

- 1. Did RIFDI prepare you to teach in a rural setting? Please describe.
  2. What were the benefits of teaching in a rural setting?
  3. What were the challenges of teaching in a rural setting?

1. We know that RIFDI participants were given protected time to do the program. How did that work for you?
2. Would you recommend this program to others at your facility?
3. Do you have any suggestions for improving the program?
4. Anything else you’d like to add?
5. IF APPLICABLE: is there anyone else from your cohort you think we should speak with about RIFDI?

**Structured Probes**

*We use structured probes frequently to follow up on any responses to the main questions on the guide. Use* ***verbatim*** *participant language – for example, if they say the program was “hard,” say “tell me more about it being hard,” NOT “tell me more about it being difficult, challenging,” etc.*

*What do you mean by _____________?*

*Walk me through ___________.*

*Tell me more about ___________.*

*Give me an example of ___________.*

*Tell me about a time when ___________.*

*What were the benefits of ___________?*

*What were the challenges of ___________?*

*What was the impact of ___________?*

*How did you learn about_________?*

*Did you receive any support for __________?*

*What could be improved about __________?*

*Who ___________?*

*When ___________?*

*Where ___________?*

**Post-Program Interview Guide**

**Introduction: First interview**

Hello [participant name],

My name is [interviewer name] and I am from the [location] VA. (*Also on the call is [second interviewer name]*.) [I am/We are] part of an evaluation team working with OAA to understand the experiences of staff who participated RIFDI.

Our goal is to understand the long-term impact of RIFDI on your professional development and to collect information to help improve the program.

We really appreciate your willingness to be interviewed. The call will take approximately 20 minutes.

Taking part in this interview is voluntary and your responses will be strictly confidential. We also want to make clear that even though we’re working with OAA and the RIFDI team, we only present them deidentified findings. In other words, you will not be identified in any reports or presentations.

Finally, in order to make sure we capture all the information you share, we would like to record this call. A recording and transcript will be created. [*If the participant has their video on, please let them know that the recording will include video unless they turn it off. Give them time to turn off their video if they prefer to record audio only*.] The recording and transcript files will be transferred to a secure server that only the evaluation team has access to.

Is this okay with you?

Before I start recording, do you have any questions for me (us)?

**[Hit record button.]** Okay, to confirm, I’m starting the recording with –

- Audio and transcript. Is this okay with you?
- Audio-video and transcript. Is this ok with you?

**Introduction: Subsequent interview (if already completed program-end interview)**

Hi [participant name],

Thanks for agreeing to speak with me *(us)* again. Last time, our goal was to understand your experience participating in RIFDI. For this interview, we’re interested in learning about what you’ve been up to since completing RIFDI and understanding the longer-term impact of the program.

The call will take approximately 20 minutes.

Like before, taking part in this interview is voluntary and your responses will be strictly confidential. In other words, you will not be identified in any reports or presentations. In order to make sure we capture all the information you share, I’d like to record this call. An audio recording and transcript will be created. The recording and transcript will be transferred to a secure server that only the evaluation team has access to. You may ask us to pause or stop recording at any time.

[*If the participant has their video on, please let them know that the recording will include video unless they turn it off. Give them time to turn off their video if they prefer to record audio only*.]

Is this okay with you?

Before I start recording, do you have any questions for me (*us*)?

[**Hit record button**.] Okay, to confirm, I’m starting the recording with –

- Audio and transcript. Is this okay with you?
- Audio-video and transcript. Is this ok with you?

# Main Questions (First and subsequent interview)

1. Tell me about what you have been doing since completing the RIFDI program.
2. What impact did RIFDI have on you, if any?
3. Has RIFDI impacted your job? (If yes, say more about that).
   1. [Probe if necessary]: have you experienced any job changes since completing RIFDI (like a promotion or new role)?
   2. How has RIFDI impacted your feelings about your job?
4. Do you continue to use the skills you gained from RIFDI in your clinical practice and teaching? *Probe for elaboration.*
5. What has been the impact of RIFDI on training programs in your area, if any? *Probe for elaboration.*
   1. IF NEEDED: What was the impact of your project, if any?
   2. IF NEEDED: What about training programs in rural settings?
6. Have you kept in touch with people you met in RIFDI?
7. What is needed to support RIFDI graduates in the long-term?
8. Anything else you’d like to add?

Those are all the questions I have – do you have any questions for me?

Thanks so much for your time and all the information – this is very helpful.

# Structured Probes (First and subsequent interview)

*We use structured probes frequently to follow up on any responses to the main questions on the guide. Use* ***verbatim*** *participant language – for example, if they say the program was “hard,” say “tell me more about it being hard,” NOT “tell me more about it being difficult, challenging,” etc.*

*Walk me through ___________.*

*Tell me more about ___________.*

*Give me an example of ___________.*

*What do you mean by _______________?*

*Tell me about a time when ___________.*

*What were the benefits of ___________?*

*What were the challenges of ___________?*

*How did you learn about_________?*

*Did you receive any support for __________?*

*What could be improved about __________?*

*What was the impact of ___________?*

*Who ___________?*

*When ___________?*

*Where ___________?*
